# Smartwatch-enabled early detection and treatment of cardiomyopathy induced by premature atrial contractions: a case report

**DOI:** 10.1093/ehjcr/ytaf151

**Published:** 2025-04-15

**Authors:** Nibras Soubh, Eva Rasenack, Helge Haarmann, Markus Zabel, Leonard Bergau

**Affiliations:** Department of Cardiology and Pneumology, University Medical Center in Göttingen (UMG), Robert-Koch-Str. 40, 37085 Göttingen, Germany; Department of Cardiology and Pneumology, University Medical Center in Göttingen (UMG), Robert-Koch-Str. 40, 37085 Göttingen, Germany; Department of Cardiology and Pneumology, University Medical Center in Göttingen (UMG), Robert-Koch-Str. 40, 37085 Göttingen, Germany; Department of Cardiology and Pneumology, University Medical Center in Göttingen (UMG), Robert-Koch-Str. 40, 37085 Göttingen, Germany; Department of Cardiology and Pneumology, University Medical Center in Göttingen (UMG), Robert-Koch-Str. 40, 37085 Göttingen, Germany

**Keywords:** Premature atrial contractions, Supraventricular extrasystole, Ablation therapy, Heart failure, Wearable devices, Case report

## Abstract

**Background:**

Arrhythmia-induced cardiomyopathy (AiCM) is an increasingly recognized cause of heart failure. Atrial fibrillation and premature ventricular contractions are common causes of AiCM. Further, the use of wearable technology in arrhythmia detection is increasing steadily. In this case, we report on a patient suffering from AiCM as a result of frequent premature atrial contractions (PACs) detected by a wearable device.

**Case summary:**

A 34-year-old male, with no prior medical history, presented after his smartwatch detected frequent ‘heart arrhythmias.’ An initial electrocardiogram showed sinus rhythm with frequent PACs. Further evaluation revealed left ventricular dysfunction [left ventricular ejection fraction (LVEF) 35%]. Non-invasive tests ruled out coronary artery disease. Although the patient was initially asymptomatic, his condition worsened with a decline in LVEF to 15% despite optimal medical therapy. High-resolution 3D mapping identified the ectopic focus in the left inferior pulmonary vein, and radiofrequency ablation successfully eliminated the PACs. At the six-month follow-up, the patient was symptom-free with a recovered LVEF of 50%.

**Discussion:**

This case highlights the potential for frequent PACs to induce AiCM. Although PACs are typically considered benign, in this case, the high PAC burden led to significant left ventricular dysfunction. The successful resolution of the patient’s symptoms and the improvement in heart function after radiofrequency ablation demonstrate the reversibility of AiCM caused by PACs if treated early. Importantly, this case underscores the emerging role of wearable technologies, like smartwatches, in the early detection of arrhythmias, enabling timely intervention to prevent the progression of heart failure.

**Learning points**

- Wearable technology can play an important role in the early detection of arrhythmias and facilitate timely interventions, leading to improved patient outcomes.
- Arrhythmia-induced cardiomyopathy should be considered in the evaluation of newly diagnosed heart failure. Arrhythmia-induced cardiomyopathy can also occur in patients with non-sustained premature atrial contractions (PACs). Ablation therapy is a feasible and effective treatment option for patients with frequent PACs and suspected arrhythmia-induced cardiomyopathy.

## Introduction

Arrhythmia-induced cardiomyopathy (AiCM) is an increasingly recognized aetiology of heart failure, characterized by myocardial dysfunction triggered or worsened by sustained or frequent arrhythmias. This dysfunction is often reversible with effective control or elimination of the arrhythmia. The most common arrhythmias associated with AiCM are atrial fibrillation and premature ventricular contractions (PVCs).^1^ In recent years, wearable technologies, particularly smartwatches, have become instrumental in the early detection of arrhythmias due to their capacity to continuously monitor heart rhythm and capture electrocardiogram (ECG) traces during symptomatic episodes. This case report describes a young patient with AiCM induced by frequent non-sustained premature atrial contractions (PACs), which are mostly considered benign. Interestingly, the arrhythmia was first detected by the patient’s smartwatch, enabling early diagnosis and timely management of both the PACs and the related cardiomyopathy.

## Summary figure

**Figure:**
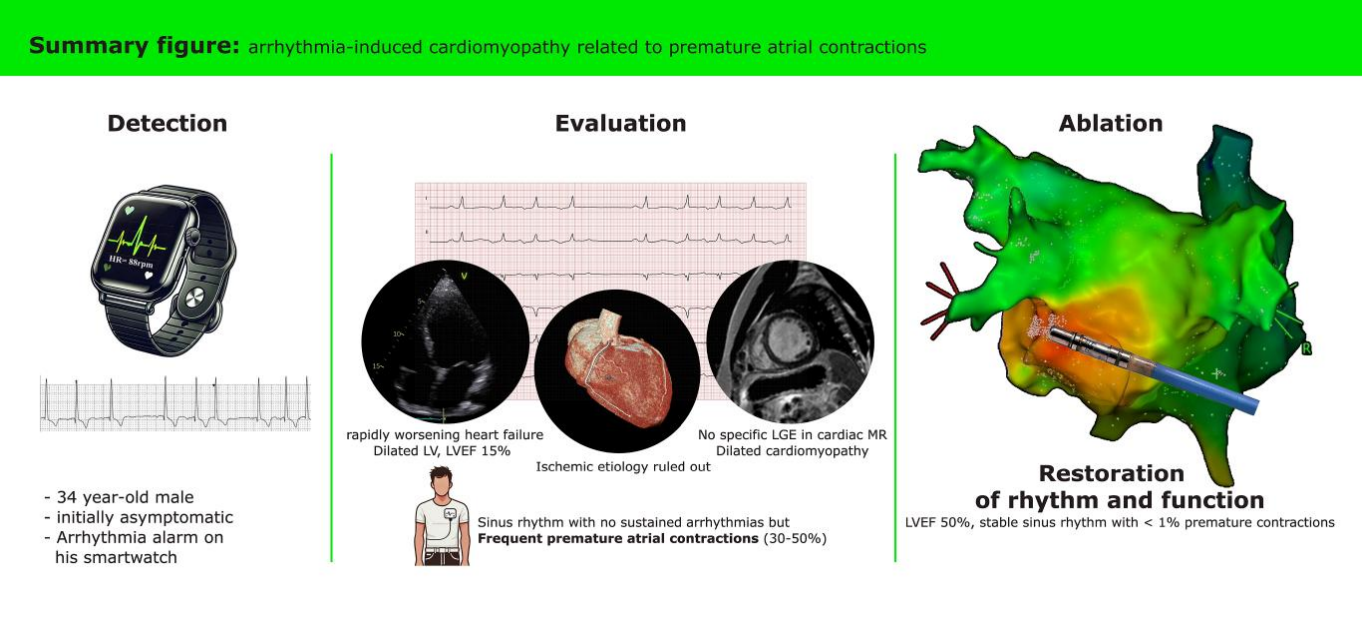


## Case presentation

**Figure 1:**
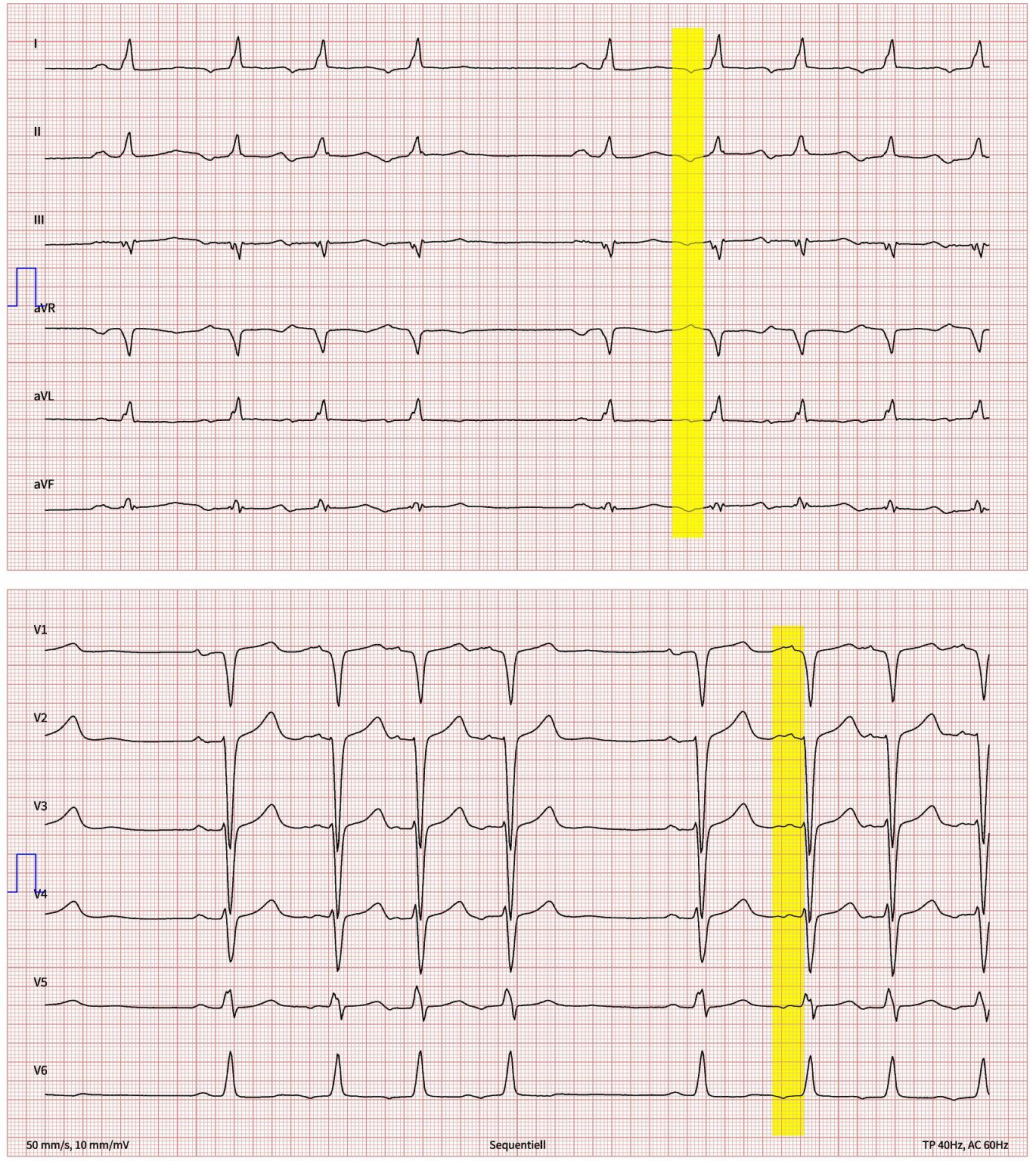
Twelve-lead ECG showing sinus rhythm with frequent premature atrial contractions. The P-wave morphology is highlighted.

**Figure 2:**
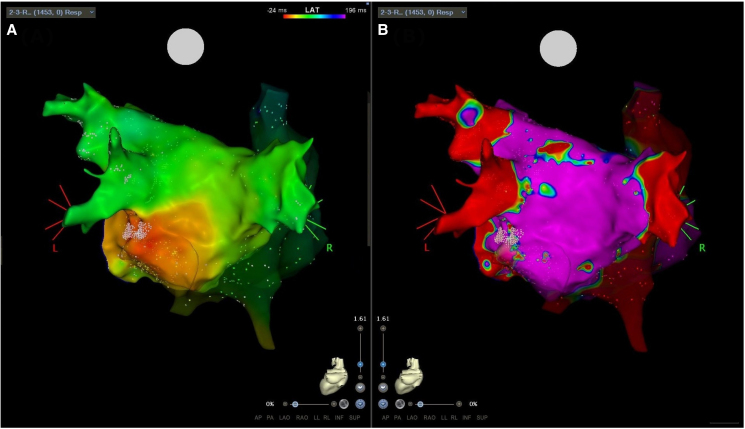
3D high-density map of both atria in posterior–anterior view. (*A*) shows the activation map of the premature atrial contraction revealing the ectopic focus (earliest activation site) in the area of left inferior pulmonary vein (red area), the ablation in this area (white points) terminated the arrhythmia. (*B*) shows the bipolar voltage map of the left atrium.

**Figure 3:**
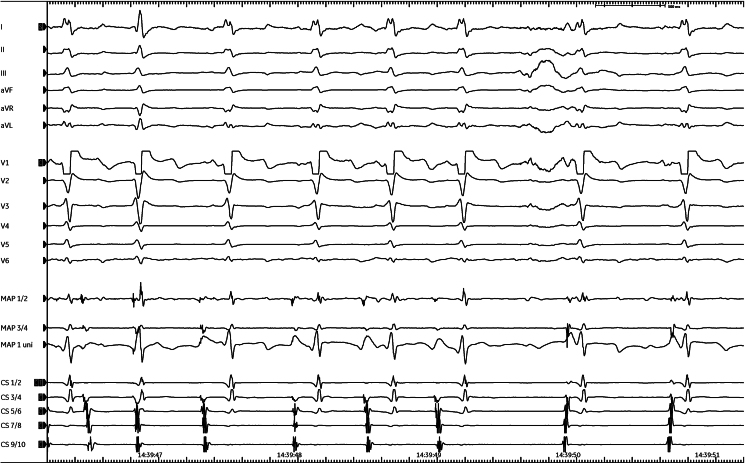
EP-system recordings after starting ablation showing local firing as response to ablation and termination of the premature atrial beats. MAP 1-2/3-4/uni represents the bipolar and unipolar recordings from the tip of the ablation catheter; CS 1-10: recordings from the decapolar coronary sinus (CS) catheter.

A 34-year-old male with no prior medical history consulted his primary care physician after his smartwatch detected ‘heart arrhythmias’. At this time, the patient was asymptomatic, denying palpitations, chest pain, or exertional dyspnoea. A 12-lead ECG revealed normal sinus rhythm with narrow QRS complex and frequent PACs. Further evaluation by a cardiologist showed a significantly reduced left ventricular ejection fraction (LVEF) of 35%, attributed to global hypokinesia, severely enlarged left ventricular end-diastolic diameter (LVEDD = 69 mm), normal left atrial volume index (LAVI = 32.6 mL/m^2^), and no valvular abnormalities. The patient was placed on guideline-directed medical therapy, including bisoprolol, valsartan/sacubitril, empagliflozin, and spironolactone, and scheduled for follow-up. A few months later, the patient presented to the emergency room with worsening palpitations and chest pain. After ruling out acute myocardial infarction, diuretics were prescribed, and the patient was referred to our advanced heart failure outpatient clinic for further evaluation. The patient had no family history of heart disease and denied smoking, alcohol, and drug abuse. A cardiac MRI revealed a dilated left ventricle with severely reduced systolic function due to global hypokinesia (LVEF = 15%) without specific late gadolinium enhancement patterns. Coronary artery disease was ruled out using cardiac CT angiography. Multiple ECGs and Holter monitor recordings confirmed an unusually high burden of PACs (30%–50%) and frequent irregular supraventricular runs with a maximum duration of 25 beats. The mean heart rate was in the upper-normal range (99 beats per minute), and no sustained atrial arrhythmias (>30 s) were documented. The supraventricular beats were monomorphic (P wave positive in V1, negative in I, II, III, aVF, and aVL) (*Figure 1*), and not interpolated, with a few wide QRS complexes (1%–2%) suspicious for aberrant conduction. The patient was scheduled for an electrophysiologic study using a 3D mapping system. The local-activation-time mapping using a multipolar high-density (HD) mapping catheter identified the ectopic focus of the PACs in the region of the left inferior pulmonary vein (*Figure 2*) (Video 1). The application of radiofrequency energy induced immediate local firing, and the arrhythmia terminated within few seconds under ablation (*Figure 3*). After ablation, a stable sinus rhythm was observed, and no further PACs occurred despite provocation with isoproterenol. No further atrial arrhythmias were inducible at the end of the procedure, and no additional ablations were needed. At the six-month follow-up visit, the patient reported complete resolution of symptoms and significantly improved exercise capacity. An echocardiogram revealed recovery of the systolic and diastolic left ventricular function, with an LVEF of 50%, HFA-PEFF-Score of 1 point, and LVEDD of 53 mm. Holter monitoring showed only 145 PACs per day (<1%), with no supraventricular runs. We made a shared decision with the patient to de-escalate heart failure treatment by discontinuing spironolactone while maintaining the remaining medications, with a follow-up scheduled in six months.

## Discussion

We are reporting a case of a young, initially asymptomatic, patient with frequent non-sustained supraventricular contractions (PACs), which were first detected by his smartwatch. Shortly after the initial diagnosis, the patient developed a rapidly worsening heart failure reaching an LVEF of 15%. Non-invasive tests ruled out specific cardiomyopathies and coronary artery disease. His ECG tracings were all remarkable for frequent PACs without other sustained arrhythmias. The ectopic focus could be identified and successfully ablated using HD mapping and radiofrequency ablation, which led to a significant improvement of his LVEF and elimination of his symptoms. The association between sustained atrial arrhythmias, particularly atrial fibrillation, and cardiomyopathy is meanwhile well-established.^2^ However, the role of non-sustained PACs in the development of cardiomyopathy remains unclear. Preliminary evidence from animal studies suggests that paced atrial bigeminy may reduce left ventricular function,^3^ though this has not yet been demonstrated in large human studies.^4^ The pathophysiological mechanisms by which PACs may induce left ventricular dysfunction are still not fully understood. Unlike PVCs, where the ventricular dyssynchrony is thought to play a central role in PVC-induced AiCM,^1^ PACs typically conduct without aberrancy and are not associated with ventricular dyssynchrony. Increased heart rate, contraction irregularity, stroke volume variations, loss of atrioventricular synchrony, and atrial remodelling are believed to mediate the PAC-induced ventricular cardiomyopathy.^5^ Yet, due to limited clinical evidence, the exact PAC burden required to initiate these pathological processes remains unclear.

The largest study investigating the effect of PACs on left ventricular dysfunction was a single-centre retrospective study that included 846 patients and found no significant correlation between PACs and left ventricular cardiomyopathy.^4^ However, it is worth noting that the mean PAC burden in this study was only 1.7%, with just 62 patients exceeding a 5% burden. In contrast, case reports of PAC-induced cardiomyopathy often describe patients with a much higher burden, typically exceeding 30%,^5–7^ suggesting that a high PAC burden may be necessary to induce clinically significant heart failure. Furthermore, the potential role of genetic predisposition, location of ectopic focus, or coupling interval in the development of cardiomyopathy remains an open question. Randomized controlled trials have firmly established the positive prognostic benefits of catheter ablation in patients with both atrial fibrillation and heart failure.^8,9^ A small clinical trial has shown that radiofrequency ablation of non-sustained PACs in patients without structural heart abnormalities is feasible and results in a significant symptomatic relief.^10^ In our case, the ablation of the PACs not only eliminated symptoms but also led to a remarkable improvement in LVEF, from 15% to 50%. We performed only limited focal ablation and decided against complete pulmonary vein isolation, as multiple Holter monitor and smartwatch recordings showed no evidence of atrial fibrillation or flutter.

Wearable technologies, especially smartwatches, are playing an increasingly important role in the detection of atrial fibrillation.^11^ To the best of our knowledge, this is the first reported case of PAC-related AiCM initially detected by a wearable device. This case underscores the expanding capabilities of wearable devices, not only in identifying common arrhythmias like atrial fibrillation but also in the early detection of subtler conditions like PACs. The patient’s journey from the initial detection of PACs via a smartwatch to the successful treatment using high-definition 3D mapping and radiofrequency ablation illustrates how advances in technology are reshaping medical practice and providing earlier detection, timely interventions, and improved outcomes.

## Lead author biography


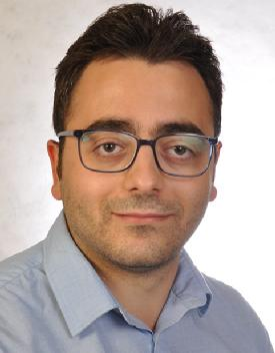


Dr Nibras Soubh studied medicine at Damascus University, Syria, and continued his training in Internal Medicine and Cardiology in Germany. He is currently an electrophysiology fellow at the University Medical Center Göttingen, Germany. His research focuses on novel ablation technologies for atrial fibrillation and the potential applications of artificial intelligence in cardiac electrophysiology.

## Data Availability

The data underlying this article are available in the article.
